# Parasitic wasp responses to symbiont-based defense in aphids

**DOI:** 10.1186/1741-7007-10-11

**Published:** 2012-02-24

**Authors:** Kerry M Oliver, Koji Noge, Emma M Huang, Jaime M Campos, Judith X Becerra, Martha S Hunter

**Affiliations:** 1 Department of Entomology, University of Georgia, Athens, GA 30605, USA; 2 Department of Biological Production, Akita Prefectural University, Akita, Japan 010-0195; 3 Department of Entomology, University of Arizona, Tucson, AZ 85721, USA; 4 Department of Biosphere 2, University of Arizona, Tucson, AZ 85721, USA

## Abstract

**Background:**

Recent findings indicate that several insect lineages receive protection against particular natural enemies through infection with heritable symbionts, but little is yet known about whether enemies are able to discriminate and respond to symbiont-based defense. The pea aphid, *Acyrthosiphon pisum*, receives protection against the parasitic wasp, *Aphidius ervi*, when infected with the bacterial symbiont *Hamiltonella defensa *and its associated bacteriophage APSE (*Acyrthosiphon pisum *secondary endosymbiont). Internally developing parasitoid wasps, such as *A. ervi*, use maternal and embryonic factors to create an environment suitable for developing wasps. If more than one parasitoid egg is deposited into a single aphid host (superparasitism), then additional complements of these factors may contribute to the successful development of the single parasitoid that emerges.

**Results:**

We performed experiments to determine if superparasitism is a tactic allowing wasps to overcome symbiont-mediated defense. We found that the deposition of two eggs into symbiont-protected aphids significantly increased rates of successful parasitism relative to singly parasitized aphids. We then conducted behavioral assays to determine whether *A. ervi *selectively superparasitizes *H. defensa*-infected aphids. In choice tests, we found that *A. ervi *tends to deposit a single egg in uninfected aphids, but two or more eggs in *H. defensa*-infected aphids, indicating that oviposition choices may be largely determined by infection status. Finally, we identified differences in the quantity of the trans-β-farnesene, the major component of aphid alarm pheromone, between *H. defensa*-infected and uninfected aphids, which may form the basis for discrimination.

**Conclusions:**

Here we show that the parasitic wasp *A. ervi *discriminates among symbiont-infected and uninfected aphids, and changes its oviposition behavior in a way that increases the likelihood of overcoming symbiont-based defense. More generally, our results indicate that natural enemies are not passive victims of defensive symbionts, and that an evolutionary arms race between *A. pisum *and the parasitoid *A. ervi *may be mediated by a bacterial symbiosis.

## Background

Insects and other arthropods are frequently infected with heritable bacterial symbionts [1]. Theory predicts that these strictly inherited microbes must confer net benefits or manipulate host reproduction to invade and persist in host populations [2-4]. While documented fitness benefits of symbionts have been primarily nutritional, a number of recent studies have reported protective effects of symbiont infection, suggesting that defense against natural enemies may be another major route facilitating invasion of heritable symbionts into host populations [5-7]. Insect symbionts of diverse bacterial lineages have been shown to provide substantial protection against fungal pathogens [8,9], viruses [10,11], predators [12,13], parasitoids [14-17] and parasitic nematodes [18]. Despite increasing awareness of the roles of these bacteria in protecting hosts, little is known about natural enemy responses to this line of defense. Just as herbivorous insects have evolved strategies to overcome plant chemical defenses, natural enemies may employ strategies to counter symbiont-based defense.

The pea aphid, *Acyrthosiphon pisum*, has become a model for characterizing the effects of infection with symbionts [19]. In addition to carrying the obligate nutritional symbiont, *Buchnera aphidicola*, this aphid is often infected with additional facultative bacteria, called 'secondary' symbionts, which have been found to mediate important ecological interactions. These effects include thermal tolerance [20,21], resistance to fungal pathogens [9] and parasitoid wasps [14]. In the latter case, *A. pisum *acquires partial to complete immunity to parasitism by the common parasitoid wasp *Aphidius ervi *via infection with the γ-proteobacterial symbiont *Hamiltonella defensa *[14,22]. In no-choice assays, wasps readily parasitized *H. defensa-*infected aphids, but failed to complete development in resistant aphids [14]. While the details of the protective mechanism of *H. defensa *remain unclear, a bacteriophage called APSE (*Acyrthosiphon pisum *secondary endosymbiont) is required to produce the protective phenotype, suggesting a key role for eukaryotic toxins carried by the phage [23,24]. Among examined lines, *A. pisum *lacking *H. defensa *(or those carrying *H. defensa *that lack APSE) are highly susceptible to parasitism by *A. ervi*, while lines carrying APSE-infected *H. defensa *receive partial to complete protection depending on *H. defensa *strain and associated phage haplotype [22-24].

Given the low probability of survival for wasp eggs laid in aphids infected with *H. defensa *and APSE, wasps should benefit from strategies that allow them to avoid or overcome symbiont-mediated defense. One potential strategy involves the use of dose-dependent factors associated with the number of oviposition events in a particular host [25]. Endoparasitoid wasps such as *A. ervi *typically employ both maternal and embryonic factors to counter host defenses and create an environment suitable for wasp development [26]. During oviposition, for example, female *A. ervi *wasps inject a γ-glutamyl transpeptidase-containing venom, which targets and degenerates pea aphid ovarioles, leading to a reduction in aphid fecundity and presumably enhancing resource availability for developing wasps [27,28]. Also, fast-growing polyploid cells called teratocytes dissociate from the serosal membrane surrounding the wasp embryo and circulate within the aphid hemocoel during larval development [29]. In *A. ervi*, teratocytes are known to synthesize and release two proteins that are likely involved in redirecting host resources from aphids to developing wasps: a fatty acid binding protein (*Ae*-FABP), and an extracellular enolase (*Ae*-ENO), which may degrade aphid tissue [30,31]. If an *A. ervi *female oviposited twice in a single host, she would then double the quantity of both venom and teratocytes present in the host, potentially increasing the likelihood of successful parasitism in symbiont-defended hosts. *Aphidius ervi *is a solitary endoparasitoid; regardless of the numbers of eggs deposited, only one parasitoid will emerge from an aphid host. Nonetheless, in a host with more than one egg, the surviving wasp may gain an advantage in the host-parasitoid conflict from additional complements of venom and teratocytes.

In this study we examined potential wasp responses to symbiont-mediated defense in *A. pisum*. We conducted experiments to determine if superparasitism, defined here as the deposition of more than one egg, increases the likelihood of successful parasitism in symbiont-defended aphids. We also conducted a choice test to determine whether female *A. ervi *wasps' oviposition behavior differs when presented with both *H. defensa *infected and uninfected *A. pisum*. Lastly, we analyzed the volatile signature of *H. defensa-*infected and uninfected aphids as wasps could potentially exploit volatile differences occurring between symbiont infected and uninfected hosts to modify oviposition behavior when faced with symbiont-protected hosts. In bark beetles, for example, natural enemies use symbiont-derived volatiles to locate hosts [32,33].

## Results

### Is superparasitism a strategy to overcome symbiont-based defense?

**Figure 1:**
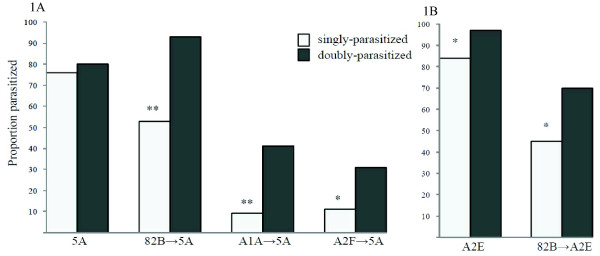
**Effect of superparasitism on successful parasitism among *H. defensa*- protected lines of*A. pisum***. Light columns are singly-parasitized aphids, dark columns superparasitized aphids (* *P *≤0.01, ** *P *< 0.001).

We conducted parasitism assays of singly versus doubly parasitized aphids to determine if the deposition of two eggs (that is, superparasitism) versus one increased the likelihood of successful parasitism of aphids harboring defensive symbionts. We examined the effects of superparasitism on a range of experimental lines that varied in aphid genotype, infection status and *H. defensa *strain (Table 1). As expected from earlier studies [14,22], aphid lines infected with *H. defensa *received significant protection when singly parasitized by *A. ervi *relative to uninfected aphids sharing the same clonal background (Figure 1A, B, Table 2A. The amount of protection varied significantly (Table 2B) and depended upon *H. defensa *strain, with the 82B strain, which carries APSE-2 (encoding *cdtB *toxin) (Table 1), conferring moderate protection in both aphid backgrounds (5A and A2E), and the two strains (A1A, A2F) carrying APSE-3 (encoding YD-repeat toxin) conferring high levels of protection (Figure 1). We also found that superparasitism (the deposition of two eggs) resulted in higher rates of successful parasitism compared to single parasitism (deposition of one egg) in all lines infected with *H. defensa *(Table 2C, Figure 1A, B). The increase in successful parasitism was observed in both aphid backgrounds and among all three *H. defensa *strains, which vary with respect to the phage haplotypes (APSE-2 and 3) carried. Between the two uninfected clones (5A and A2E) there was no overall effect of superparasitism on rates of successful parasitism in uninfected clones (Table 2C), but when considered individually the results appeared to vary. In uninfected clone 5A, double-parasitism did not lead to a significant increase in the proportion successfully parasitized, while it did in clone A2E. This difference between single and double parasitism in clone A2E is unexpected if double-parasitism acts to only overcome *H. defensa-*associated resistance. However, upon closer examination, there was no increase in the number of mummies produced following double parasitism of A2E (t-test *P *= 0.60), just fewer surviving aphids overall, which skewed the proportion of successfully parasitized aphids. In all *H. defensa*-infected strains, on the other hand, we found significant increases in the number of mummies when aphids were superparasitized. When doubly-parasitized, we found a 45% increase in mummies in line 82B→5A, a 200% increase in mummies in A1A→5A, a 157% increase in A2F→5A, and a 32% increase in 82B→A2E. Overall, we did not detect significant increases in aphid mortality in superparasitized versus singly parasitized cohorts across all treatments (ANOVA F_1,119 _= 0.25 *P *= 0.61) or when restricted to *H. defensa*-infected lines (ANOVA F_1,79 _= 2.21, *P *= 0.14). Together this indicates that the significant increases in successful parasitism rates of superparasitized aphids in *H. defensa*-infected aphids was due primarily to greater production of mummies and not increased aphid mortality. Finally, in our logistic regression model, we found a significant interaction between infection status (infected vs. uninfected) and parasitism treatment (one vs. two oviposition events) among all lines (Likelihood effects ratio test, df = 5, Χ^2 ^= 11.8, *P *= 0.047) indicating that superparasitism led to a greater increase in successful parasitism in *H. defensa*-infected lines compared to uninfected lines.

**Table 1:** Experimental lines of *A.pisum *used in this study

*A. pisum *line	*H. defensa* strain	APSE- haplotype	phage toxin	Clone color	Collection Info: aphid clone (C) or symbiont donor (D)
5A	none			pink	C: Madison, WI 1999

82B→5A	82B	APSE2	*cdtB*	pink	D: Cayuga Co., NY 2000

A1A→5A	A1A	APSE3	YD-repeat	pink	D: Logan UT 2004

A2F→5A	A2F	APSE3	YD-repeat	pink	D: Logan UT 2004

A2E	none			green	C: Logan UT 2004

82B→A2E	82B	APSE2	*cdtB*	green	D: Cayuga Co., NY 2000

**Table 2:** Logistic regression analyses: regression equation is Y = β_0 _+ β_1 _+ β_2 _

A. **Resistance effects of infection with *H. defensa *relative to uninfected control**		
**assay**	**Regression equation**	***P-*value**

5A vs. 82B→5A	Y = 0.66 - 0.49^Hd^	*P *= 0.02

5A vs. A1A→5A	Y = -0.57 - 1.73^Hd^	*P *= 0.0001

5A vs. A2F→5A	Y = -0.57 - 1.72^Hd^	*P *= 0.0001

A2E vs. 82B→A2E	Y = 0.69 - 0.90^Hd^	P = 0.001

**B**. Variation in resistance among *H. defensa *strains in common background 5A		

A2F vs. 82B vs. A1A	Y = -1.13 + 1.3^82B ^-0.16^A1A^	*P *= 0.001

**C**. effects of superparasitism on rates of successful parasitism		

**Assay**	**Regression equation**	***P-*value**

5A (uninfected)	Y = 1.28 + 0.13^DP^	*P *= 0.57

82B→5A (*H. defensa *+ APSE-2)	Y = 1.44 + 1.27^DP^	*P *= 0.001

A1A→5A (*H. defensa *+ APSE-3)	Y = -1.34 + 0.97^DP^	*P *= 0.0005

A2F→5A (*H. defensa *+ APSE-3)	Y = -1.55 + 0.73^DP^	*P *= 0.004

A2E (uninfected)	Y = 2.54 + 0.94^DP^	*P *= 0.01

82B→A2E (*H. defensa *+ APSE-2)	Y = 0.34 + 0.55^DP^	*P *= 0.008

All treatments	Y = 0.35 + 0.40^DP^	*P *< 0.0001

All *H. defensa*-infected	Y = -0.26 + 0.46^DP^	*P *< 0.0001

All uninfected	Y = 1.71 + 0.32^DP^	*P *= 0.07

DP, double-parasitism; Hd, *H. defensa*

### Number of eggs and teratocytes in singly versus doubly parasitized aphids

We dissected parasitized aphids to verify that the number of observed parasitism events (single vs. double) corresponds to the number of eggs deposited in a single aphid host. In singly-parasitized *A. pisum *we found one egg in 86% (69/80) aphids (two eggs in 0/80, no eggs in 11/80). In doubly-parasitized aphids we detected two eggs in 83% aphids (66/80) (one egg in 13/80, no eggs 1/80). In the aphids containing one fewer *A. ervi *egg than expected, it is unclear whether no egg was deposited or if we just did not find it. Regardless, these results indicate that one egg is typically deposited per parasitism event. We also examined whether double parasitism results in an extra complement of teratocytes. In uninfected line 5A, we found roughly twice the number of teratocytes in doubly parasitized aphids (N = 8, mean 45.8, 95% CI = 41.7 to 49.8) relative to aphids parasitized once (N = 8, mean 23.1, 95% CI = 19.1 to 27.2) indicating one complement of teratocytes is associated with each oviposition event. In singly parasitized, *H. defensa*-infected aphids (line 82B→5A), however, we found a significant reduction (ANOVA F_1, 179 _= 71.2, *P *< 0.0001) in the number of teratocytes in aphids containing a dead or moribund larva (N = 104, mean = 16.0, 95% CI = 14.8 to 17.2) compared to aphids with a healthy larva (N = 77, mean = 23.7, 95% CI 22.4 to 25.1) suggesting that a healthy complement of teratocytes may be important for wasp survival in a symbiont-protected host. We also observed that some teratocytes from this *H. defensa*-infected line (82B→5A) were irregular in shape, compared to those collected from uninfected aphids sharing the same genetic background (line 5A).

### Do wasps selectively superparasitize *H. defensa*-infected aphids?

**Figure 2:**
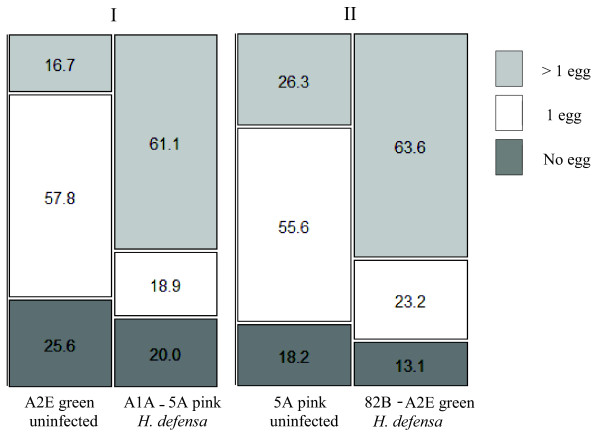
**Distribution of parasitism 'choices' between *H. defensa*-infected and uninfected aphids**. Numbers inside boxes are percentages which sum to 100%. Dark grey boxes indicate percentage of aphids not parasitized, white the number singly parasitized, and light grey the number superparasitized. N = 11 for each treatment.

To determine if female *A. ervi *superparasitize *H. defensa*-infected aphids at a greater frequency than uninfected aphids, we conducted behavioral assays to monitor oviposition behavior of wasps. The pea aphid exhibits a pink-green color polymorphism, and we used clone color as a visual marker of infection status. We conducted two assays, each with an infected clone of one color/genotype paired with an uninfected clone of the other color/genotype. In choice tests, we found that female *A. ervi *wasps tended to lay a single egg in uninfected aphids while depositing more than one egg in *H. defensa-*infected aphids (Figure 2). The distribution of parasitism 'choices' (that is, no egg, one egg, > 1 egg) differed significantly between the green uninfected (A2E) and the pink *H. defensa*-infected (A1A→5A) (trial one, likelihood ratio test, Χ^2 ^= 43.5, *P *< 0.0001), as well as between the pink uninfected (5A) and green *H. defensa*-infected lines (trial two, likelihood ratio test, Χ^2 ^= 30.2, *P *< 0.0001). The majority of *H. defensa-*protected aphids, of both pink (A1A→5A) and green (82B→A2E) clones, were superparasitized, while the majority of uninfected *A. pisum *(5A = pink, A2E = green) were singly parasitized. The distribution of parasitism choices did not vary between green (A2E) and pink (5A) uninfected clones (likelihood ratio test, Χ^2 ^= 3.3, *P *= 0.2), nor between green *H. defensa*-infected (82B→A2E) and pink *H. defensa*-infected (A1A→5A) lines (likelihood ratio test, Χ^2 ^= 1.8, *P *= 0.4). Together these results indicate that oviposition choices in these assays were largely determined by infection status rather than clone color.

### Compound identification and quantification using GC-MS

**Figure 3:**
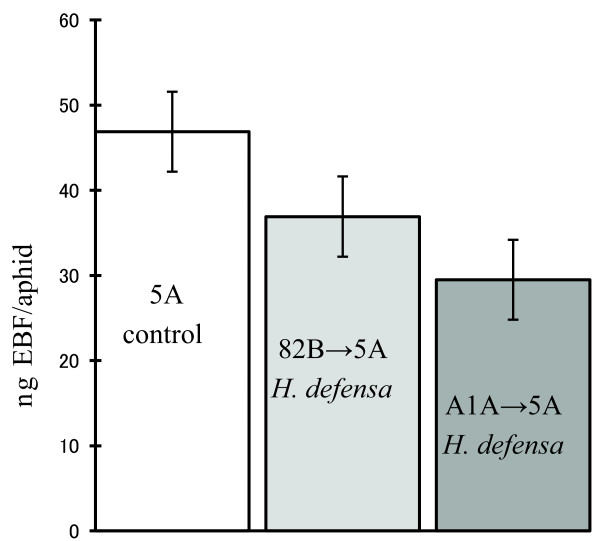
**Amounts of *trans*-β-farnesene (EBF) per aphid in *H. defensa*-infected versus uninfected *A. pisum*.** Bars = SE, N = 11 each line.

In whole aphid extracts, we identified significant differences in the quantity of *trans*-β-farnesene (EBF), between *H. defensa*-infected and uninfected aphids sharing the same genetic background (Figure 3). EBF, a volatile sesquiterpene, is the major component of the aphid alarm pheromone [34]. Aphids secrete alarm pheromone from cornicles when attacked. The pheromone alerts nearby aphids, which respond by walking away from the source of the pheromone, or by dropping from the food plant [35-37]. The alarm pheromone may also serve as a short-range host location cue used by both predators and parasitoids (reviewed in [38]). In uninfected aphid clone 5A, we detected 46.9 (95% CI = 37.2 to 56.5) ng of EBF/aphid compared to 36.9 ng EBF/aphid (95% CI = 26.3 to 46.5) in *H. defensa*-infected line 82B→5A and 29.5 (95% CI = 19.8 to 39.1) ng EBF/aphid in *H. defensa*-carrying line A1A→5A (ANOVA F_2, 30 _= 3.46 = 0.04). All lines share the same aphid background so differences are due to infection status. We also used t-tests to evaluate each *H. defensa*-infected line relative to the uninfected control and found that EBF quantities in line A1A→5A were significantly reduced relative to the control (N = 11, t-test, *P *= 0.015), but that line (82B→5A) showed a non-significant reduction (N = 11, *P *= 0.18) relative to the control.

## Discussion

Recent studies indicate that many insects are protected by heritable symbionts [7], yet little is known about how natural enemies respond to symbiont-based defenses. We report here that the parasitoid wasp, *A. ervi*, can partially overcome *H. defensa*-mediated protection in *A. pisum *by superparasitizing aphids (Figure 1, Table 2). Further, we find in choice assays that *A. ervi *can discriminate between *H. defensa*-infected and uninfected *A. pisum *and selectively superparasitizes infected aphids, while laying single eggs in uninfected hosts (Figure 2). Taken together, these results indicate that *A. ervi *has likely evolved successful strategies to partially counter symbiont-based defenses employed by *A. pisum*. Counter-strategies to overcome defense on the trophic level below are well known. Herbivorous insects have devised strategies for countering plant-based defenses (for example, [39-41]), and predators and parasitoids may overcome herbivore behavioral [42,43] and chemical defenses as well. This report provides evidence that natural enemies are sometimes able to counteract symbiont-based defense. Recent reports have documented: 1) the evolution of increased *A. ervi *virulence when faced with symbiont-carrying resistant *A. pisum *lines [44] and 2) genotypic variation in the parasitoid *Lysiphlebus fabarum*'s ability to successfully parasitize *H. defensa*-infected *Aphis fabae *(black bean aphid) [16]. The experimental design of these studies, however, does not allow conclusive partitioning of increased virulence towards symbiont- versus host-based defensive factors, but these reports do indicate that parasitic wasps likely have additional mechanisms for overcoming symbiont-based resistance.

Consistent with previous reports, we found that aphids infected with *H. defensa*, when singly parasitized, received moderate to high levels of protection from attack by *A. ervi*, depending upon strain [22]. We also found that the same strain of *H. defensa *(82B) in two backgrounds (A2E and 5A) conferred similar levels of protection, indicating little symbiont X aphid interaction [22]. When doubly-parasitized, however, we found that the likelihood of successful parasitism of *H. defensa-*infected aphids increased in every instance, in both pink (5A) and green (A2E) aphid backgrounds and among all three *H. defensa *strains (82B, A1A and A2F). Strains A1A and A2F carry the YD-repeat toxin-encoding phage variant ASPE-3 and strain 82B carries the *cdtB*-encoding APSE-2 [23]. Not only does superparasitism increase successful parasitism in symbiont defended hosts, but wasps gain a relatively greater benefit of superparasitism when attacking *H. defensa *infected aphids compared to uninfected aphids. Thus, superparasitism appears to be an effective counter strategy to both phage variants hitherto detected in N. American populations in *A. pisum*. One caveat regards the possibility that wasps emerging from successfully superparasitized aphids are not fit enough to reproduce themselves. While we did not explicitly examine wasp fitness, we did verify that adult wasps emerge from superparasitized hosts and bear no obvious abnormalities. A previous study in this system found slight increases in dry weight, and no differences in developmental time, in wasps emerging from superparasitized aphids relative to singly parasitized *A. pisum *[45], indicating that superparasitism does not generally lead to reductions in wasp fitness. In future experiments we will examine costs associated with superparasitism as a strategy to overcome defensive symbionts.

The mechanisms underlying the increase in successful parasitism of superparasitized aphids are not known, but the additional maternal and embryonic factors found in superparasitized hosts may contribute. In aphids infected with 82B→5A, dead or moribund larvae appear four to five days post parasitism - long after teratocytes have dissociated from the extra-embryonic membrane [46]. We confirmed that an additional complement of teratocytes was present in doubly-parasitized aphids. We also found reductions in the number and irregularities in the morphology of teratocytes deposited in *H. defensa *infected aphids suggesting a possible interaction. Polyploid teratocytes are believed to be involved in redirecting resources from aphid to wasp development [28,29]. Fewer, or defective, teratocytes may prevent the creation of an environment suitable for wasp development. The extra complement of teratocytes in superparasitized aphids may shift the balance in favor of successful parasitoid development.

Some authors have suggested that the likelihood of one solitary parasitoid surviving host encapsulation responses may increase when multiple eggs are deposited in a single host -the so-called 'multiple target hypothesis' (for example, [47,48], rev. in [25]). While there are some accounts that host encapsulation responses are not as effective when faced with multiple parasitism events (for example, [49-51]), Vinson [52] reviewed the evidence for the multiple target hypothesis and concluded that target proliferation is probably not generally a viable strategy for endoparasitoids in overcoming host encapsulation defenses. While the aphid encapsulation response is very weak in our system, the notion of multiple parasitoid targets, especially teratocytes, diluting the effectiveness of symbiont-based defenses, may resurrect this hypothesis for cases when protective symbionts are present.

We found that not only does superparasitism increase the odds of successful parasitism, but that *A. ervi *females appear to discriminate between *H. defensa*-infected aphids and uninfected aphids (Figure 2) and preferentially superparasitize symbiont-defended aphids. This indicates that wasps likely employ superparasitism as a strategy to overcome symbiont-based defense. In choice tests, females were more likely to lay single eggs in uninfected aphids, but more than one egg in *H. defensa *infected aphids. Since only one wasp can complete development in a single aphid, self-superparasitism (more than one egg laid by the same mother) by solitary endoparasitoids like *A. ervi *has generally been considered maladaptive, yet wide-ranging conditions have been proposed under which this strategy may be adaptive [51]. If the net fitness of offspring is higher under conditions of superparasitism compared to single-parasitism then selection may favor the sacrifice of an additional egg for a parasitoid such as *A. ervi*, which is limited by their time to find hosts rather than the number of eggs they have [25,53]. *Aphidius ervi *has a large number (96 to 567) of small, yolk-free eggs during adulthood (for example, [54,55]). While we did not compare the net fitness of wasps when singly parasitizing versus superparasitizing hosts, we did find large increases in the numbers of mummies (32 to 200%) produced by superparasitism in each of the *H. defensa*-infected lines. This sharp increase in successful parasitism combined with the relatively low expected cost of *A. ervi *eggs suggests that superparasitism may produce net benefits. Thus, superparasitism may be an adaptive strategy when faced with symbiont-defended hosts.

We found that levels of trans-β-farnesene (EBF) were lower in *H. defensa*-infected aphids (Figure 3), and differences in the abundance of this volatile compound are a possible basis for wasp discrimination between infected and uninfected hosts. EBF is the major component of aphid alarm pheromone and is secreted from the cornicles (siphunculi). EBF in aphids has been found to serve as a short-range (due to high reactivity with ozone) attractant to a wide range of natural enemies, including both predators and parasitoids (rev. [38]). Cornicle secretions, although not specifically EBF, from *A. pisum *have been reported to be a contact kairomone for *A. ervi *[56]. Aphids release alarm pheromone when confronted with natural enemies, which often leads to escape behaviors, such as dropping from the host plant [36,37], and inducing the production of winged morphs [57]. In groups of symbiont-defended aphids, the release of lower amounts of EBF could be adaptive, as dropping from the plant, or dispersing is risky [58] while staying put may be a better strategy if one is likely to survive a parasitoid attack. A recent report found that *A. pisum *infected with *H. defensa *exhibited fewer escape behaviors and reduced aggressiveness relative to uninfected counterparts [59], consistent with reduced levels of EBF. More generally, heritable variation in escape behavior among *A. pisum *clones has been reported [60]; and heritable symbiont influences on EBF titers could explain some of this variation.

In addition to documenting parasitoid behavioral responses to symbiont-based defense in herbivores, it is also remarkable that aphid secondary symbionts appear to be detectable by foraging parasitoids. Oviposition by *Aphidius ervi*, and aphidiine parasitoids in general, is extraordinary in its speed; the parasitoid faces the aphid, coils its abdomen underneath its head and thorax so that it too faces forward, and appears to strike the aphid with the ovipositor at the tip of its abdomen, generally laying an egg in that instant. Aphidiine wasps are not generally known to lay two eggs with a single strike [45,61], so superparasitism involves a second strike and oviposition. Detection of contact or even internal compounds likely involve sensilla on the ovipositor [62] presumably after contact chemicals have been detected and evaluated. That detection of the host's condition can occur during this very short interval has been shown by several authors [61,63,64]; female *A. ervi *were shown to avoid parasitism of aphids already containing a parasitoid egg. We show that the levels of EBF are generally lower in symbiont-defended relative to uninfected *A. pisum*. Whether EBF is the compound used to discriminate between symbiont-defended and uninfected aphids, however, is not certain. Infection with *H. defensa *and other symbionts has been shown to alter the pool of metabolites in *A. pisum *[65], and these changes may also serve as host selection cues. It would be interesting, however, if symbiont-defended aphids use reduced EBF to increase the threshold for dropping behavior, but parasitoids have co-opted the signal for their own benefit.

## Conclusions

Recent reports indicate that a wide range of insects are protected from natural enemies via infection with heritable symbionts, yet little is known about natural enemy responses to symbiont-based protection [7]. Here we report that the parasitic wasp *A. ervi *discriminates between symbiont-defended and undefended (that is, uninfected) aphids, and modifies its oviposition behavior when faced with protective symbionts in ways that increase the likelihood of successful parasitism. Wasps selectively deposit two or more eggs in symbiont-defended hosts, even though only one wasp can complete development within a single aphid. The discovery of wasp behavioral responses to symbiont-based defense in aphids sets the stage for further investigations of the coevolutionary dynamics of the host *A. pisum *and its parasitoid *A. ervi*, as mediated by a bacterial symbiosis. In general, we expect that the study of natural enemies that are confronted with defensive symbionts will yield insights into various counter-strategies to circumvent or overcome the protection these symbionts provide.

## Methods

### Aphids, heritable bacteria and experimental lines

*Acyrthosiphon pisum *(Hemiptera: Aphididae) is a phloem-feeding polyphagous pest of herbaceous legumes [66] that was introduced to North America from Europe around 1870. The pea aphid reproduces asexually, except in the autumn, when a shorter photoperiod induces the production of sexual morphs, which mate and produce diapausing eggs. By mimicking long-day length conditions (16:8 L:D) in the laboratory, clonal lineages can be maintained indefinitely. This aphid also exhibits a pink-green color polymorphism, with pink morphs encoding a carotenoid desaturase not present in green morphs [67]. Each *A. pisum *clonal line used in these experiments was comprised of descendants of a single parthenogenetic female maintained on *Vicia faba *(fava bean) at 20°C +/- 1°C, and 16:8 L:D in a biological incubator.

In this study, we used experimental lines created by microinjection for a previous study [22], which comprise combinations of three *H. defensa *strains in two *A. pisum *clonal backgrounds (Table 1). We use the term *uninfected *to refer to aphids (or clonal lines) that are uninfected with any secondary symbionts, but still retain *Buchnera*. We routinely screened lines with diagnostic PCR to ensure expected infection status as in Oliver *et al. *[22]. In our laboratory colonies the vertical transmission rate of *H. defensa *approaches 100%, and infections established by microinjection have proven stable in cultures for up to 10 years. We also performed a diagnostic fingerprinting technique (intersequence simple repeats or ISSR) to verify aphid genotype [68].

### Parasitoids

The common, solitary endoparasitoid *A. ervi *(Haliday) (Hymenoptera: Braconidae) was released in North America to control *A. pisum *populations. The adult female deposits an egg inside a suitable aphid nymph. After approximately 24 hrs (at 20°C) the egg hatches and the resulting wasp larva develops within a living aphid for about one week. The parasitoid eventually kills the aphid, consumes the host viscera, and causes the aphid cuticle to transform into the characteristic 'mummy', in which the wasp pupates. A free-living adult *A. ervi *wasp emerges from the mummy. In the current study, the wasp culture was maintained in the laboratory at 20°C +/- 1°C and 16L:8D, on an *A. pisum *clone (5A) uninfected with *H. defensa*.

### Number of eggs and teratocytes in singly versus superparasitized aphids

We dissected aphids approximately six hours (held at 20°C) after parasitism in 1X PBS (phosphate-buffered saline) solution and searched for *A. ervi *eggs with the aid of a Leica MZ6 stereomicroscope (Wetzlar, Germany) to verify that single and double parasitism events (N = 80, each) result in one and two eggs, respectively, deposited per aphid host. To determine if superparasitism results in a double complement of teratocytes, we dissected singly- and doubly-parasitized third-instar aphids (N = 8 each, uninfected line 5A) approximately 96 hours (20°C) after parasitism in 1X PBS to estimate teratocyte counts per host. We also investigated potential interactions between *H. defensa *and teratocytes by examining the number of teratocytes approximately 96 hours (20°C) after parasitism in singly parasitized aphids containing either healthy (resistant N = 77) or dead/moribund (susceptible, N = 104) wasp larvae in aphid line 82B→5A.

### Does superparasitism overcome symbiont-based defense?

Each replicate of protection assays consisted of cohorts of 10 second-instar *A. pisum *being either singly- or doubly- parasitized by *A. ervi *in a Petri dish, placed on a potted *V*. *faba *plant in a cup cage, and then held in a biological incubator at 20°C +/- 1°C and 16L:8D. We conducted 10 replicates of each treatment. Eclosion rates (adult wasps emerging from mummies) approach 100% for both singly and doubly-parasitized aphids. Since mummies are sessile (that is, easy to count accurately), and a suitable proxy to estimate parasitism rates, we counted the numbers of mummies and surviving aphids after 10 days to determine rates of successful parasitism. We conducted two assays. The first assay examined the effect of superparasitism on experimental lines 5A, 82B→5A, A1A→5A, allowing us to examine effects on two different *H. defensa *strains with different phage haplotypes and associated toxins (Table 1). In assay 2, we examined the effects of superparasitism on experimental lines A2E and 82B→A2E, allowing us to examine effects in a distinct *A. pisum *background. Parasitism assays were conducted at least 40 generations after artificial infection. Koga *et al. *[69] reported that detrimental effects of novel infections with *S. symbiotica *on *A. pisum *had attenuated by eight months post-infection. We analyzed the proportion of successfully parasitized aphids in a logistic regression (that is, computing log odds of mummification) framework testing for effects of infection status and parasitism treatment as well as calculating the interaction term between infection status and parasitism treatment. All statistical analyses were performed using JMP 9.02 software (SAS, Cary, North Carolina, USA).

### Do wasps selectively superparasitize *H. defensa*-infected aphids?

To determine if female *A. ervi *superparasitize *H. defensa*-infected aphids at a greater frequency than uninfected aphids, we conducted behavioral assays to monitor oviposition behavior of wasps. In each section of a three-way divided Petri dish we placed a second, third and fourth instar nymph of each color morph (for example, uninfected green clone = A2E, and *H. defensa*-infected pink clone A1A→5A) for a total of 18 aphids in each dish. The use of a subdivided dish, three instars, and two color morphs allowed us to identify every individual aphid and observe the number of times each aphid was parasitized. We also reversed the color scheme for half of the replicates, using the uninfected pink clone (5A) and *H. defensa*-infected green clone (82B→A2E) to account for the possibility that parasitoids preferentially prefer to attack one color morph. For each replicate of the assay, we introduced a single, mated *A. ervi *female into the dish and we recorded the number of times that each individual aphid was parasitized. We allowed wasps to forage for 30 minutes or to lay a maximum of 24 eggs (whichever came first), to increase the likelihood that most aphids were examined as potential hosts. Wasps that attacked no aphids in the first five minutes after introduction to the arena were removed and replaced with another female. The experiment was conducted 'blind' as the observer was unaware which clone (green or pink) in each trial was *H. defensa*-infected. We exposed 378 aphids to wasps and recorded more than 500 attacks in 10 to 11 trials of each combination. We analyzed the proportion of aphids that were a) not parasitized, b) singly parasitized, or c) parasitized two or more times using a likelihood ratio test contingency analysis with clone color (pink or green), infection status (*H. defensa*-infected or not), and the individual wasps as explanatory variables. Variation among individual wasps, which were never re-used, did not contribute significantly to the model, and this variable was removed from the model for subsequent analyses.

### Gas chromatography (GC) and mass spectrometry (MS) to identify volatile differences between *H. defensa*-infected and uninfected aphids

To determine whether there were detectable differences in the volatile signature of *H. defensa-*infected and uninfected aphids, replicate samples of five aphids of each type were collected in a glass tube, and were extracted with 100 μl of dichloromethane with 5 ng/μl of 1-dodecene as an internal standard (IS), for five minutes. We analyzed a two microliter sample using GC-MS carried out by an Agilent 6890N gas chromatograph linked to an Agilent 5975B mass spectrometer operated at 70 eV using a HP-5MS capillary column (Agilent Technologies (Santa Clara, California, USA), 30 m × 0.25 mm ID, 0.25 μm in film thickness) with helium carrier gas at a velocity of 1.2 ml/minute in splitless mode. The oven temperature was held at 60°C for two minutes and programmed to increase at 10°C/minute from 60°C to 300°C and then held at 300°C for five minutes. The injector temperature was maintained at 200°C and the detector temperature at 300°C. One compound, *trans*-β-farnesene (EBF), varied significantly among treatments. We verified the identity of this compound by comparing its GC retention time and mass spectrum with those of an authentic standard (Sigma-Aldrich, St Louis, Missouri, USA)). To quantify the amount of EBF in each sample, we performed GC analysis with a flame ionization detector (FID), using a J & W DB-5MS capillary column (Agilent Technologies, Santa Clara, California, USA), 25 m × 0.32 mm ID, 0.52 μm in film thickness) under the same conditions as those for GC-MS, except that the velocity of helium carrier was 2.0 ml/minute. To prepare the calibration curve for EBF, six known concentrations of the authentic standard were used (0.5 to 5 ng/μl). The peak area of the compound was divided by that of the IS, and then a calibration curve for the compound was prepared using the peak area ratio and the known concentrations. The concentrations of the compound in samples were determined by comparing their peak area ratio with the one found in the calibration curve. The amounts of EBF recorded were normally distributed and we conducted an ANOVA and t-tests to compare means among/between treatments as well as to determine (1 - α) 95% confidence intervals using JMP 9.02 software (SAS, Cary, North Carolina, USA.

## Abbreviations

APSE: *Acyrthosiphon pisum *secondary endosymbiont**; **EBF: *trans*-β-farnesene; FID: flame ionization detector; ISSR: intersequence simple repeats**; **PBS: phosphate-buffered saline

## Competing interests

The authors declare that they have no competing interests.

## Authors' contributions

KMO, JXB and MSH designed the study. KMO, KN, EMH and JMC performed the research. KMO and MSH analyzed the data and wrote the paper. All authors read and approved the final manuscript.
